# Flow cytometric detection of leukemic stem cells in Acute Myeloid Leukemia: current status and future directions

**DOI:** 10.3389/fphar.2025.1724473

**Published:** 2025-12-10

**Authors:** Mariam M. Youssef, Christopher Y. Park

**Affiliations:** Pathology, New York University Grossman School of Medicine, New York, NY, United States

**Keywords:** AML-acute myeloid leukemia, LSC-leukemic stem cells, flow cytometry, MRD-measurable residual disease, therapeutic targeting biomarkers

## Abstract

The assessment of measurable residual disease (MRD) plays a critical role in acute myeloid leukemia (AML) treatment response evaluation and prognosis. However, current AML MRD detection by flow cytometry (FC) is limited in sensitivity due to immunophenotypic variability, similarities to normal hematopoietic stem/progenitor cells, and the lack of stable leukemia-associated immunophenotypes. A significant proportion of AML patients classified as MRD-negative by FC eventually relapse, likely due to the persistence of therapy-resistant leukemic stem cells (LSCs) that are not sensitively detected by routine clinical flow panels. Flow cytometry panels designed to detect LSC antigens, while promising, face challenges like immunophenotypic heterogeneity across AML subtypes, lack of standardized marker panels across laboratories, and limited validation. Here, we summarize the current state of FC-based LSC detection in AML, discussing commonly used markers, immunophenotypic variability, assay setup challenges, and we review recent clinical studies on LSC assessment, outlining their main findings and implications for prognosis and MRD integration. We also consider advances in spectral flow cytometry for improved LSC detection.

## Introduction

Acute myeloid leukemia (AML) is an aggressive cancer of hematopoietic stem and progenitor cells (HSPCs), affecting approximately 20,000 individuals annually in the United States (Dohner et al., 2015). Despite therapeutic advances, prognosis remains poor with a 60%–70% mortality rate (National Cancer Institute, 2024), primarily due to post-remission relapse.

Measurable residual disease (MRD) detection is essential in guiding treatment for several hematologic malignancies (Short et al., 2020). In B-lymphoblastic leukemia (B-ALL), MRD detection by flow cytometry (FC) achieves high sensitivity, often below 0.01% (Theunissen et al., 2017), and holds prognostic significance (Verbeek and van der Velden, 2024). Similarly, in multiple myeloma FC–based MRD detection is highly sensitive to 0.001%, offering strong prognostic value and thus routinely incorporated into response assessments (Medina-Herrera et al., 2023; Flores-Montero et al., 2017). However, in AML, MRD detection remains challenging, primarily due to patient-to-patient immunophenotypic variability and the lack of stable, aberrant leukemia-associated immunophenotypes (van der Linde et al., 2023). The sensitivity of FC for MRD detection in AML is typically limited to around 0.1%, as estimated in clinical studies (Blachly et al., 2022; Hanekamp et al., 2020). A significant proportion of AML patients deemed MRD-negative by FC ultimately relapse (Ivey et al., 2016; Terwijn et al., 2013), indicating that current MRD assays fail to detect low-levels of clinically relevant AML blasts (Thakral et al., 2022), supporting the notion that therapy-resistant clones that include functional leukemic stem cells (LSCs) enriched for self-renewal, dormancy, and drug resistance (Ivey et al., 2016; Terwijn et al., 2013), persist after treatment and drive disease recurrence (Joshi et al., 2019). Detecting LSCs by FC remains a significant challenge due to their low frequency, antigen heterogeneity, and overlap with normal HSPCs (Terwijn et al., 2010; Srinivasan Rajsri et al., 2023). Moreover, the great majority of reported LSC antigens have not been incorporated into routine MRD panels in clinical flow cytometry laboratories.

In this review, we summarize current knowledge on the application of FC to identify LSCs in AML. We discuss the phenotypic characteristics of LSC-enriched fractions, commonly used surface markers, and how these profiles vary across AML molecular subtypes. We also highlight supporting evidence from functional assays such as xenotransplantation, address key technical considerations (e.g., required cell numbers, optimal sample processing), and explore the clinical implications and limitations of FC-based LSC detection in the context of MRD monitoring. Finally, we consider how advances in spectral (full-spectrum) FC are expanding the possibilities for deeper, more precise immunophenotyping of rare and heterogeneous LSC populations.

### Immunophenotypic identification of leukemic stem cells

The search for methods to identify LSC antigens has been based on the longstanding paradigm that AML blasts are transformed HSPCs expressing novel antigens or antigenic patterns that reflect the normal sequence of antigen expression in normal HSPCs. Efforts to identify normal human hematopoietic stem cells (HSCs) led to reports in 1991 that CD34^+^CD38^−^ cells contain primitive progenitors (Terstappen et al., 1991), and in 1992, the addition of CD90 enriched for cells capable of multilineage engraftment in immunodeficient mice (Baum et al., 1992). This foundational work helped establish the commonly accepted phenotype of human HSCs as CD34^+^CD38^−^CD90+Lineage^−^, although subsequent studies further refined this phenotype and showed that HSCs are CD45RA- (Majeti et al., 2007) and can be further enriched by identifying CD49f+ cells in the CD34^+^CD38^−^CD90^+^CD45RA-Lineage^-^ fraction (Notta et al., 2011). In 1994, Lapidot et al. demonstrated that rare AML cells with a CD34^+^CD38^−^ phenotype could initiate leukemia in immunodeficient mice (Lapidot et al., 1994), establishing this population as enriched for LSC activity. The CD34^+^CD38^−^ compartment has since served as the primary focus for LSC identification in AML, although subsequent studies demonstrate that other blast fractions harbor LSC activity, albeit at reduced frequencies (Eppert et al., 2011; Ng et al., 2016). This raises important questions: Are LSCs defined by a uniform phenotype? Do distinct LSC populations arise in different AML subtypes? And do LSCs represent cell states without normal counterparts in normal hematopoietic development?

In a pivotal study, Goardon et al. demonstrated that LSCs are not confined to a single immunophenotype but can be found in two hierarchically related compartments (Goardon et al., 2011). In the majority of AML cases, they identified both a CD34^+^CD38^−^CD90^−^CD45RA^+^ population resembling lymphoid-primed multipotent progenitors (LMPP-like LSCs) and a more differentiated CD34^+^CD38^+^CD123^+^/lowCD110^−^CD45RA^+^ population resembling granulocyte-monocyte progenitors (GMP-like LSCs). Crucially, only LMPP-like LSCs were able to generate GMP-like cells *in vitro* and *in vivo*, but not the reverse, indicating a developmental hierarchy and suggesting greater stemness potential of the LMPP-like subset (Goardon et al., 2011).

Although CD45RA positivity has been proposed to distinguish LSCs from HSCs (Cloos et al., 2018; Boyer et al., 2018), CD45RA expression alone may not be sufficient to effectively distinguish LSCs from normal progenitors since AML blasts frequently lose CD90 expression (Holden et al., 1995; Inaba et al., 1997; Kozii et al., 1997). A study observed that LMPPs express CD38 at an intermediate level between CD38^−^ stem cells and CD38^+^ progenitors, recommending a narrower CD38 negative gate to reduce misclassification (Kersten et al., 2016). Therefore, using CD45RA in conjunction with other aberrant LSC markers may enhance the specificity and accuracy of LSC identification.

Altough the CD34^+^CD38^−^ compartment is frequently enriched for LSC activity in CD34^+^ AMLs, LSCs have also been described in AMLs lacking CD34 expression, which show <1% CD34 expression and comprise approximately 20% of all AMLs (van der Pol et al., 2003; Zeijlemaker et al., 2015; van Rhenen et al., 2005). Therefore, alternative markers such as CD117 have been used to enrich for stem-like cells (Quek et al., 2016). In NPM1-mutated AML, which is often associated with low CD34 expression, studies have shown that some cases harbor LSCs exclusively within the CD34^−^ fraction, while others contain LSCs in both the CD34^+^ and CD34^−^ fractions (Taussig et al., 2010; Quek et al., 2016). More recently, venetoclax resistance in monocytic AML has been attributed to a distinct monocytic LSC population (CD34^−^CD14^−^CD11b^−^CD36^−^), which is immunophenotypically distinct from previously described CD34^−^ LSC subsets and characterized by reduced BCL2 dependence (Pei et al., 2023). Together, these observations underscore the immunophenotypic heterogeneity of AML LSCs and highlight the limitations of relying on single-marker gating. To address this, additional markers such as CD99 (Chung et al., 2017). CD133 (Reuvekamp et al., 2025a) CD32 (Ho et al., 2016), CLL-1 (Larsen et al., 2012), CD244 (Quek et al., 2014) have been explored in CD34^−^ AML, either individually or in combination with CD38 (Ho et al., 2016). The most commonly reported markers that enrich for LSCs in AML are listed in Table 1.

**TABLE 1:** Review of surface markers that enrich for leukemic stem cells in acute myeloid leukemia.

Marker	Definition/Biology	Findings	References
CD123 (IL-3Rα)	α-subunit of IL-3 receptor	Highly expressed on CD34^+^CD38^−^ AML LSCs; absent/low on normal HSCs but present on committed progenitors. Expression is associated with adverse prognosis. Reportedly upregulated after therapy in regenerative marrow. Ongoing clinical trials of CAR-T cells and antibody-drug conjugate targeting CD123	Goardon et al. (2011), Sarry et al. (2011), Jin et al. (2009), Vergez et al. (2011), Jordan et al. (2000)
CD45RA	Isoform of leukocyte phosphatase CD45	Highly expressed on AML LSCs, particularly those with a LMPP-like phenotype. Absent on normal HSCs but present on some normal committed progenitors. Improves discrimination between LSCs and normal HSCs	Goardon et al. (2011), Boyer et al. (2018), Kersten et al. (2016)
CLL-1 (CLEC12A/CD371)	C-type lectin-like inhibitory receptor	Broadly expressed on AML LSCs in both CD34^+^ and CD34^−^ disease; xenograft studies confirm leukemia-initiating capacity. Absent on HSCs, but present on normal monocytes and granulocytes. Ongoing development of CAR-T cells and antibody-drug conjugates to target CLL-1	Larsen et al. (2012), van Rhenen et al. (2007b), Joudinaud and Boyer (2021), Noordhuis et al. (2010)
TIM-3 (CD366)	Immune checkpoint receptor	Upregulated on AML LSCs and implicated in maintaining self-renewal. Expression is associated with relapse risk and poor survival. Absent on HSCs, but present on activated immune cells (e.g., T cells, monocytes), which may limit therapeutic targeting	Jan et al. (2011), Kikushige et al. (2015), Darwish et al. (2016), Akashi (2015)
CD44	Hyaluronan receptor; adhesion molecule	Expressed on both AML LSCs and normal HSCs, but more highly and functionally upregulated on LSCs. Expression is associated with chemoresistant leukemic cells. Blockade eradicates LSCs in xenograft models, though expression on HSCs limits specificity	Jin et al. (2006), Das et al. (2024), Gutjahr et al. (2021), Charrad et al. (2002)
CD96	Ig superfamily receptor; binds CD155/PVR	Expressed on some AML LSCs, with expression associated with poor prognosis. Absent on HSCs but present on activated immune cells, reducing specificity	Hosen et al. (2007), Du et al. (2015)
CD47	Integrin-associated protein; “don’t-eat-me” checkpoint	Overexpressed on AML LSCs, associated with inferior survival. Also broadly expressed on normal HSCs and blasts, which limits specificity	Majeti et al. (2009), Majeti et al. (2008), Sallman et al. (2019)
CD99	Cell adhesion/signal protein	Highly expressed on AML LSCs and implicated in leukemogenesis; enriched at relapse. Absent on HSCs. Therapeutically targetable *in vitro*, but expression on other normal tissues may limit specificity	Chung et al. (2017)
IL1RAP	Co-receptor for IL-1α/β and IL-33 signaling	Highly expressed on AML LSCs, with higher levels in FLT3-mutated cases. Minimal on HSCs. Attractive target in preclinical models, but expression on some normal progenitors and non-hematopoietic tissues may limit therapeutic specificity	Zhang et al. (2024), Metois et al. (2025), Askmyr et al. (2013)
GPR56 (ADGRG1)	Adhesion GPCR.	Highly expressed on AML LSCs and is associated with poor prognosis. Also expressed on normal HSCs, which limits its specificity as a therapeutic target.	Ng et al. (2016), Pabst et al. (2016), Daria et al. (2016), Saito and Morishita (2015)
CD33	Sialic acid–binding Ig-like lectin (Siglec)	CD33 is strongly expressed on AML LSCs. Also expressed at low levels on normal progenitors, and its expression can change after therapy, which limits its reliability as an MRD marker	Zeijlemaker et al. (2016), Terwijn et al. (2014)
CXCR4 (CD184)	Chemokine receptor for CXCL12	Overexpressed on AML LSCs, mediating bone marrow niche retention and promoting survival. Associated with poor prognosis. Blockade of CXCR4 mobilizes LSCs into circulation, which may increase their susceptibility to chemotherapy. Also expressed on normal HSCs, which limits specificity	Biondi et al. (2021), Roboz et al. (2018), Maganti et al. (2020)
CD70	TNF ligand binding CD27	Aberrantly expressed on AML LSCs and often upregulated at relapse. Blocking CD70 reduces leukemic engraftment in preclinical models. Absent on HSCs, but inducibly expressed on activated immune cells, which limits specificity. Under investigation in antibody- and CAR-T–based therapeutic trials	Ochsenbein et al. (2019), Riether et al. (2017), Marques-Piubelli et al. (2024), Hinterbrandner et al. (2017)
CD36	Fatty acid transporter	Marks metabolically distinct, quiescent AML LSCs; expression is enriched after therapy and associated with poor survival. Reported as absent on HSCs, but its role as a discriminator remains under investigation. Further studies are needed to validate its utility as an AML LSC marker	Landberg et al. (2018), Farge et al. (2023), Abacka et al. (2024), Zhang et al. (2020), Guerrero-Rodriguez et al. (2022)
CD97	Adhesion GPCR of EGF-TM7 family	Highly expressed on AML LSCs, where it supports self-renewal and is associated with poor prognosis. Minimal on HSCs. Also expressed on normal leukocytes and not yet used in clinical MRD setting or therapeutic studies	Martin et al. (2016), Martin et al. (2019)
CD93	C-type lectin receptor	Expressed on AML LSCs, particularly in MLL-rearranged AML, where it promotes proliferation. Preclinical antibody blockade reduces AML progression. Also expressed on endothelial cells and some myeloid progenitors, which may restrict specificity and therapeutic applicability	Kinstrie et al. (2020), Iwasaki et al. (2015), Jia et al. (2022), Richards et al. (2021)
CD69	Early activation antigen (C-type lectin)	Marks chemoresistant, quiescent AML LSCs, with high expression linked to poor survival. Not yet incorporated into flow MRD assays. Widely induced on activated immune cells, which limits specificity	Antony et al. (2023), Zhou et al. (2025), Qiu et al. (2022), Zhang et al. (2023)
CD200	Immunoglobulin superfamily glycoprotein	Enriches for LSCs in both CD34^+^ and CD34^−^ AML and contributes to immune suppression within the marrow microenvironment. Also expressed on normal HSCs, limiting specificity for LSC detection or therapeutic targeting	Ho et al. (2020), Coles et al. (2012), Coles et al. (2011)
CD25 (IL-2Rα)	α-chain of IL-2 receptor	Enriched on quiescent AML LSCs and associated with early treatment failure. CD25^+^ LSCs can give rise to CD25^−^ leukemic progeny, underscoring intrapopulation heterogeneity. Absent on normal HSCs at the transcriptomic level, but FC testing in healthy donors remains limited	Saito et al. (2010), Kageyama et al. (2018), Cerny et al. (2013)
CD32 (FcγRII)	Low-affinity Fc receptor	Present on LSCs in ∼30% of AML. In some cases, CD32^+^CD34^+^CD38^−^ cells initiate AML in xenografts, but in others only the CD32^−^ fraction has leukemic potential and is enriched for quiescent LSCs. Absent on normal HSCs. Enriches LSCs in certain CD34^−^ AMLs, but heterogeneous expression patterns limit its reliability as a universal marker	Ho et al. (2016), Saito et al. (2010)
CD166 (ALCAM)	Ig superfamily adhesion molecule	Upregulated in subsets of AML and enriched in LSC-like transcriptomic clusters. However, also expressed on normal HSCs, stromal cells, and endothelial cells, limiting its specificity as an AML LSC marker	Shimura-Nukina et al. (2016)
EMR2 (CD312)	Adhesion GPCR in same family as CD97	Expressed on AML blasts and LSCs, with preclinical studies suggesting it may be targetable with CAR-T approaches. Absent on HSCs but expressed on normal mature myeloid cells, which limits specificity	Unglaub et al. (2023), Xie et al. (2025)
CD133 (Prominin-1)	Stem/progenitor marker	CD133^+^ cells display LSC-like activity and can help identify LSCs in CD34^−^ AML. However, CD133 is also expressed on normal HSCs and progenitors, limiting its specificity	Reuvekamp et al. (2025a), Heo et al. (2020)
CD90 (Thy-1)	Glycoprotein stem cell marker	AML blasts are frequently CD90^−^, and the CD90^−^ fraction is enriched for leukemogenic activity. Normal HSCs are typically CD90^+^, so loss of CD90 expression may help distinguish LSCs from normal HSCs	Holden et al. (1995), Inaba et al. (1997), Kozii et al. (1997), Richards et al. (2021), Brendel et al. (1999), Buccisano et al. (2004)
CD109	GPI-anchored glycoprotein; TGF-β modulator	Identified in LSC transcriptomic signatures, with high expression correlated with poor prognosis. However, CD109 has not been widely used as a FC marker for LSCs	Wagner et al. (2019)
CD74	Invariant chain of MHC II; receptor for MIF	Upregulated in slow-cycling AML subsets and reported in pediatric AML. Explored as a potential therapeutic target, but evidence as an AML LSC marker remains limited	Menssen et al. (2024), Li et al. (2024a)
CD98	Amino acid transporter; integrin enhancer	Overexpressed in AML and implicated in LSC self-renewal and engraftment. Functional studies support its role as a therapeutic target, but it is not widely applied as a FC marker for LSC detection or MRD monitoring	Hayes et al. (2015), Bajaj et al. (2016), Reinisch and Majeti (2016)
CD26 (DPP4)	Dipeptidyl peptidase-IV enzyme	Selectively expressed on FLT3-ITD^+^ AML LSCs. Largely absent in FLT3- wildtype AML.	Herrmann et al. (2020)
CD49d (α4 integrin)	Integrin α4 subunit; forms VLA-4	Highly expressed on AML blasts, including LSC-enriched fractions, and linked to increased MRD burden and relapse risk. Therapeutically targetable but broad expression on normal immune cells and stroma limits its specificity as an LSC marker	Grenier et al. (2021), Ogana et al. (2024)
CD9	Tetraspanin family protein	Expressed on AML LSCs and enriched in the CD34^+^CD38^−^ fraction. Absent on HSCs at the transcriptomic level but detected on normal multipotent and lymphoid-primed progenitors at levels comparable to LSCs, which limits its specificity	Liu et al. (2021), Touzet et al. (2019)
CD244 (2B4)	SLAM family receptor	Frequently expressed on AML LSCs, where it supports leukemia maintenance and is associated with poor prognosis. Also expressed on normal HSPCs, monocytes, and various non-hematopoietic tissues, which limits its specificity	Haubner et al. (2019)
Lineage markers (CD2, CD7, CD11b, CD14, CD15, CD19, CD22, CD56)	Lymphoid/myeloid differentiation antigens	Frequently aberrantly expressed on AML blasts, including within the CD34^+^CD38^−^ compartment, but not specific for stemness. Absent on HSCs. Primarily useful for LAIP determination and distinguishing leukemic from normal progenitors in MRD assessment	Goardon et al. (2011), Kersten et al. (2016), Zeijlemaker et al. (2016)

### Functional validation and limitations of stemness assays

Functional validation serves as the gold standard for confirming that immunophenotypically defined cell populations truly represent LSCs. This process relies heavily on xenotransplantation into immunodeficient mice to initiate disease as well as serial engrafment in order to demonstrate self-renewal capacity. However, these assays have inherent limitations. Successful engraftment depends on multiple factors including homing efficiency, immune evasion, and responsiveness to murine cytokines. Thus, it is possible that some LSCs with true leukemogenic potential may fail to engraft due to species-specific barriers. Indeed, using xenograft models, LSC frequencies have been reported to range from as high as ∼1 in 10^4^ cells to as low as ∼1 in 10^7^ cells, underscoring both their rarity and the marked variability between patients (Sarry et al., 2011; Bonnet and Dick, 1997). This necessitates complementary approaches, such as fluorescence *in situ* hybridization (FISH), next-generation sequencing (NGS), and targeted mutational profiling, to confirm the presence of leukemia-specific cytogenetic or molecular abnormalities. Moreover, linking these features with clinical outcomes like disease-free and overall survival is crucial to establish their clinical relevance.

Exploration of alternative LSC identification strategies that do not rely solely on surface antigens has been pursued. Some studies highlight metabolic distinctions, with LSCs in the CD34^+^CD38^−^ fraction usually exhibiting lower aldehyde dehydrogenase (ALDH) activity than residual normal HSCs in AML bone marrows (Schuurhuis et al., 2013). However, AML LSCs with high ALDH activity have also been described (Hoang et al., 2015; Ran et al., 2009; Blume et al., 2018), indicating that ALDH-based discrimination is variable across subtypes and lacks the specificity to serve as a standalone marker. Other strategies attempt to detect LSCs using similar methods to HSC detection, like Hoechst-low side population (SP) cells, which are enriched for stem cell activity based on dye efflux ability (Feuring-Buske and Hogge, 2001; Moshaver et al., 2008). Given that both malignant and normal stem cells exhibit these features (Moshaver et al., 2019), distinguishing between them remains a challenge requiring additional markers to distinguish between them.

### Challenges in quantifying LSCs in clinical settings

Accurate identification of LSCs by FC is challenging due to the immunophenotypic heterogeneity of AML. Although employing extended antibody panels can improve detection, the variation in surface marker expression across different cases may require customized panel designs guided by initial diagnostic screening. Current FC-based strategies for LSC detection are limited by antibody panel variability among labs, the substantial phenotypic overlap between leukemic and normal progenitor compartments, subjective gating, and the lack of incorporation of antibodies that can reliably identify LSCs.

But what markers would constitute optimal markers for LSC identification? This involves assessing the specificity of candidate markers by comparing their expression in blasts and putative LSCs versus normal HSCs. Normal HSCs can be identified using negative control bone marrow samples or in the AML specimen, as we and others have shown the presence of residual HSCs in AML diagnostic samples (Jan et al., 2012; Chung et al., 2017). Ideally, a useful marker should show clear differences in marker expression within the CD34^+^CD38^−^ compartment, either as a distinct population (marker^+^ vs. marker^−^) or by overexpression in LSCs relative to normal HSCs. Zeijlemaker and colleagues proposed a scoring system ranking markers by their ability to provide such distinction (Zeijlemaker et al., 2016), emphasizing the importance of minimizing contamination of LSCs in the marker-negative fraction. However, marker-negative fractions cannot always be assumed to be free of LSCs. For example, analysis of GPR56 in combination with CD34 expression revealed engraftment potential in both CD34^−^ and CD34^+^ fractions, demonstrating that not all LSCs express the classical CD34^+^CD38^−^ phenotype (Pabst et al., 2016). Similarly, Saito et al. showed that CD32 is highly expressed in LSCs, yet the CD32^−^ fraction in a subset of cases also retains leukemic potential and is enriched for quiescent LSCs (Saito et al., 2010). These findings underscore the need for cautious interpretation as well as validation of markers across diverse AML subtypes.

Markers with continuous rather than bimodal expression also requires carefully defined thresholds for positivity to differentiate true aberrancy from background noise. Common approaches include using mean fluorescence intensity (MFI) ratio or fold change relative to isotype controls (Chung et al., 2017; Haubner et al., 2019), setting cutoffs at least two standard deviations from appropriate negative reference (Loghavi et al., 2021), or applying receiver operating characteristic (ROC) curves to optimize discrimination (Guy et al., 2013).

Homogeneous CD38 expression in some AML cases poses challenges for gating as a clearly defined CD38^−^ compartment may be absent. In such cases, internal controls such as residual erythroid cells or calibration beads for CD38-negative thresholds (Cloos et al., 2018; Quek et al., 2014) and monocytes (Terwijn et al., 2014) or hematogones (Ngai et al., 2025) as CD38-positive references, provide a biologically anchored framework for interpretation.

Another consideration lies in the limitation in cell number when using conventional FC. When large panels are split across multiple tubes, sufficient events may not be acquired to confidently detect rare LSCs. To overcome this, prior studies have attempted to incorporate several LSC markers into a single fluorescence channel within a one-tube assay (Zeijlemaker et al., 2016; Li et al., 2022). However, this approach does not allow the flexibility to interpret complex expression patterns of markers and nonuniform expression of lineage markers. This issue is particularly significant for rare cells, as collective assessment of populations with different expression levels and/or autofluorescence may lead to decreased sensitivity and specificity of LSC detection (Boesch et al., 2018). Furthermore, post-therapy immunophenotypic shifts within the shared channel can affect accurate LSC measurement. However, when markers are assessed separately, it requires significant effort to determine which markers are co-expressed or absent to identify a residual LSC population as subsets may be negative for certain markers yet positive for others.

Different approaches have been used to define LSC burden. Some studies calculate the proportion of LSCs relative to total WBCs (Ngai et al., 2023; Zeijlemaker et al., 2019a; Terwijn et al., 2014), and others calculating LSCs as a percentage of CD34^+^ cells or primitive compartments (Ngai et al., 2025). Regardless of the method used, LSC frequency cutoffs have been calculated based on clinical endpoints. Several studies have addressed this by correlating LSC frequency with remission and relapse outcomes. For example, a cutoff of 0.03% CD34^+^CD38^−^ LSCs linked to higher relapse risk, with higher frequencies associated with inferior remission rates and survival (Zeijlemaker et al., 2019b). In the post-therapy setting, thresholds are typically pushed closer to the limit of detection of FC, since residual LSCs are expected to be rare (Zeijlemaker et al., 2019a).

As the disease progresses, shifts in immunophenotype may occur due to clonal evolution, relapse, or treatment effect. Ideally, LSC markers should maintain stable expression after chemotherapy (Saito et al., 2010). Yet some, such as CD123 are also reported to be expressed in regenerating marrow, limiting specificity (van Rhenen et al., 2007a) Further work is also needed to clarify how these markers behave in the context of clonal hematopoiesis or myelodysplasia, and how to interpret LSC-like phenotypes in these settings. One study reported that post-remission clonal hematopoiesis can produce immunophenotypic alterations in myeloid progenitors exceeding typical regenerative patterns yet remaining distinct from the original AML, underscoring the need to avoid misclassifying such cells as MRD (Loghavi et al., 2021).

### Flow cytometry studies of LSCs in AML

Several studies have applied multiparameter FC panels combining more than one marker to distinguish LSCs from normal HSCs and assess their prognostic relevance and ability to assess MRD. Table 2 summarizes many published studies in this area, ranging from single-center explorations to cooperative trial-based investigations. Please note that not all published studies could be included due to space limitations.

**TABLE 2:** Multiparameter flow cytometry studies of leukemic stem cells in acute myeloid leukemia.

Study	Markers/panel used	Summary of findings
Hwang et al. (2012)	Panel includes: CD34, CD38, CD123, CD44, CD184	In 63 AML patients, the median proportion of CD34^+^CD38^−^ leukemic blasts was 1.3%. Among CD34^+^CD38^−^ cells, 74.6% expressed CD123, 100% expressed CD44, and 85.7% expressed CD184. Patients who achieved complete remission had significantly lower LSC proportions compared to those who did not achieve remission
Terwijn et al. (2014)	Multi-tube panel including: CD45, CD34, CD38; lineage markers (CD2, CD7, CD11b, CD19, CD22, CD56), CLL-1, CD13, CD33, HLA-DR, CD45RA. Marker choice for MRD was based on screening for >10% positivity at diagnosis	Approximately 75% of AML cases exhibited aberrant marker expression within the CD34^+^CD38^−^ compartment (e.g., CLL-1, lineage markers). Using this combined immunophenotypic and light-scatter–based approach, putative leukemic and normal stem cell populations could be distinguished in ∼47% of samples, with a median of 82% of the CD34^+^CD38^−^ fraction identified as neoplastic, and higher LSC frequencies associated with inferior clinical outcomes
Angelini et al. (2015)	Multi-tube panel including: CD34, CD117, CD38, CD123, CD25, CD99, CD7	Identified a distinct CD34^+^CD123^+^CD99^+^CD25^+^ immunophenotype that was present in ∼30–40% of FLT3-ITD AML. This population was detectable at diagnosis in some patients who later acquired FLT3-ITD relapse, suggesting it may mark high-risk subclones. The study primarily focused on genetic correlation rather than LSC phenotyping
Cheng et al. (2016)	Multi-tube panel including: CD45, CD34, CD38, lineage markers, CD33, CD123, CD133, CD44, HLA-DR	In pediatric AML, CD33 and CD123 were highly expressed on AML LSCs, and demonstrated significant differences compared with their expression on normal HSCs
Kersten et al. (2016)	Multi-tube panel including: CD45, CD34, CD38, CD45RA, CLL-1, CD33, CD123	CD45RA was the most reliable marker for identifying LSCs within the CD34^+^CD38^−^ compartment. Cases with >90% CD45RA^+^ LSCs showed higher scatter values (P < 0.001) of LSC compared to HSCs
Zeijlemaker et al. (2016) Zeijlemaker et al. (2019b)	8-color one-tube assay including: CD45, CD34, CD38, CD45RA, CD123, CD33, CD44, and a composite channel (CLL-1/TIM-3/CD7/CD11b/CD22/CD56)	Developed an 8-color one-tube LSC panel defining LSCs within the CD34^+^CD38^−^ compartment as cells positive for aberrant markers (CD123, CD33, CD45RA, or a composite channel). CD123 and CD33 were aberrant in ∼82% of cases; CD123 was reliable at diagnosis but upregulated post-therapy. CD44 was uniformly expressed on HSCs but often overexpressed in LSCs. A single channel combining multiple markers correlated well with individual markers for CD34^+^CD38^−^ LSC quantification
Freeman et al. (2018)	One-tube assay including: HLA-DR, CD45, CD123, CD56/CD2, CD34, CD117, CD7 and CD38	In younger adults, a high frequency of LSCs at diagnosis was predictive of relapse risk. Patients with an LSC load >17.2% of CD34^+^ blasts were at especially high risk
Yabushita et al. (2018)	Panel includes CD25, CD96, CD123	In a cohort of *de novo* AML patients, expression of two or more of CD25, CD96, and CD123 was associated with poorer 3-year overall survival
Haubner et al. (2019)	Multi-tube panel including: CD34, CD38 + CD33, CD123, CLL-1, TIM-3, CD244 and CD7	CD33, CD123, CLL-1, TIM-3, and CD244 were broadly expressed on AML blasts at both diagnosis and relapse, independent of genotype. Dual-marker analysis showed that CD33/TIM-3 and CLL-1/TIM-3 combinations were most specific for AML compared with normal or non-hematopoietic tissues
Zeijlemaker et al. (2019a)	7 customized 8-color tubes with backbone markers (CD34, CD38, CD45, CD19, CD13, CD14, HLA-DR) supplemented with patient-specific markers (CD33, CLL-1, TIM-3, CD133, CD123, CD96, CD22, CD44, CD7, CD2, CD36, CD11b, CD15)	Median LSC percentage of CD34-positive cases was 0.0079%. Demonstrated that LSC frequency was an independent predictor of overall survival and combining LSC burden with MRD status after remission improved risk stratification. Notably, some patients who were initially LSC-negative became LSC-positive after therapy. However, the method required multiple patient-specific tubes, limiting its practicality for routine monitoring
Petersen et al. (2021)	15-color one-tube pediatric panel: CD45, CD3, CD117, CD34, CD38, CD123, CD45RA, CLL-1, TIM-3, IL1RAP, CD25, CD93, CD99, BCL-2, viability dye	Aberrant LSC fractions within the CD34^+^CD38^−^ compartment were quantified and t-SNE was used to support analysis. The study proposed a framework for standardized pediatric LSC assessment, but data were limited
Li et al. (2022)	8-color Zeijlemaker et al. (2016) one-tube LSC assay	Compared the traditional FC-based MRD assay with the LSC-based approach by Zeijlemaker et al. in AML patients post-allogeneic transplantation. LSC marker expression within the CD34^+^CD38^−^ compartment remained stable up to 1 year after transplant. Detection of CD34^+^CD38^−^ LSCs after allografting was useful for predicting relapse, but not for leukemia-free survival or overall survival
Ngai et al. (2023)	8-color Zeijlemaker et al. (2016) one-tube LSC assay	Prospectively validated the prognostic value of LSC burden at diagnosis and after the second chemotherapy cycle in intermediate- and adverse-risk AML. Findings demonstrated that incorporating LSC quantification into clinical trial frameworks provides additional value for identifying patient subgroups with poorer outcomes beyond standard risk stratification
Das et al. (2024)	10-color single-tube: CD45, CD34, CD38, CD117, sCD3, CD45RA, CD123, CD44, CD33; composite channel with CLL-1, TIM-3, CD25, CD11b, CD22, CD7, CD56	LSCs and HSCs were identified in 88.2% and 71.6% samples, respectively. LSCs showed significantly higher expression of composite, CD45RA, CD123, CD33, and CD44 compared with HSCs. No significant differences in LSC antigen expression were observed between CD34^+^ and CD34^−^ subsets. Multiple LSC subpopulations were detected in 4.4% of cases
Reuvekamp et al. (2025b)	8-color Zeijlemaker et al. (2016) one-tube LSC assay	In older AML patients treated with hypomethylating agents, LSC positivity was associated with higher relapse rates and shorter event-free survival. All patients who were both MRD^+^ and LSC^+^ relapsed within 6 months
Zhang et al. (2025)	29-color single-tube full-spectrum FC assay. Panel includes CD45RA, CD123, CD33, CD44, CD47, TIM-3, CD133 and CLEC12A	Developed a comprehensive spectral assay enabling simultaneous MRD and LSC detection in AML. When compared with a conventional 5-tube assay analyzing 20 AML BM samples, the panel showed high concordance and reduced sample and tube requirements

### Development of guidelines for LSC quantification in the clinical setting

In 2018, a consensus document from the European LeukemiaNet (ELN) MRD Working Party addressed key challenges in MRD assessment in AML (Schuurhuis et al., 2018). As part of their recommendations, the group proposed exploration of a separate LSC panel to assess total LSC burden at any point from diagnosis to relapse. They cited the approach described by Zeijlemaker et al. (2016) (Zeijlemaker et al., 2016), which included a panel combining CLL-1, TIM-3, CD7, CD11b, CD22, and CD56 in one channel, along with CD123, CD45RA, CD44, and CD33 in the same tube. Additional markers such as CD25, CD32, and CD99 were also noted to be potentially useful.

In the 2021 ELN MRD Working Party report (Heuser et al., 2021), evaluation of LSCs in FC-based MRD assessment was identified as a priority area for the future improvement of MRD recommendations. LSCs were defined immunophenotypically as CD34^+^/CD38^-^ cells combined with aberrant markers not expressed on normal HSCs such as CD45RA, CLL-1, or CD123. The report highlighted that LSC measurements may have prognostic relevance and should be further validated in prospective clinical trials. For optimal detection, acquisition of approximately 4 million events was recommended, ideally using a single-tube assay. Building on this, the 2022 ELN international expert panel on AML diagnosis and management also acknowledged the potential prognostic relevance of residual LSCs and stressed the need for continued investigation and validation in this area (Dohner et al., 2022).

### Prognostic significance and multimodal integration of LSC and MRD assessment in AML

Recent studies report that LSC burden in AML at diagnosis and post-treatment can carry prognostic value in adult and pediatric cohorts (Zeijlemaker et al., 2019a; Hanekamp et al., 2018). High LSC frequency is associated with earlier relapse and shorter overall survival (Ngai et al., 2023; Zeijlemaker et al., 2019a; Terwijn et al., 2014). Incorporating LSC assessment into MRD monitoring modestly improves risk stratification. For example, patients with both LAIP-MRD^high^ and LSC^high^ showed significantly reduced survival than patients with only one or neither of these features (e.g., MRD^low^/LSC^high^, MRD^high^/LSC^low^, or MRD^low^/LSC^low^) (Zeijlemaker et al., 2019a). In particular subsets such as LAIP-MRD-negative intermediate-risk patients, LSC positivity may guide considerations for transplant decisions or more intensive monitoring strategies (Li et al., 2022; Ngai et al., 2023).

Beyond its prognostic value, MRD assessment is emerging as a key component of AML management by offering enabling more risk-adapted and individualized treatment strategies. Persistently positive MRD after induction or consolidation identifies patients at elevated risk of relapse who may benefit from treatment intensification, such as allogeneic stem cell transplantation or enrollment in investigational protocols, whereas MRD negativity supports continuation of standard consolidation or therapy de-escalation (Freeman et al., 2018; Heuser et al., 2021).

Together, MRD and LSC analyses provide a complementary framework for predicting relapse and refining treatment decisions. Dual assessment enhances sensitivity and specificity over either approach alone (Li et al., 2022). Moreover, persistence or re-emergence of LSCs in the post-transplant setting has been associated with relapse (Li et al., 2025), suggesting that incorporating LSC monitoring into post-transplant surveillance could be beneficial. Collectively, these advances position LSC quantification as a powerful complement to MRD, bridging biological insight with clinical practice and refining therapeutic decision-making across the AML disease course.

Molecular MRD assessment adds an important layer of prognostic refinement in AML. Leukemia-specific assays targeting stable genetic lesions, such as NPM1 mutations, PML-RARA and core-binding factor (CBF) rearrangements, or other recurrent fusions, enable sensitive detection of residual disease in certain genetically defined AML subtypes (Heuser et al., 2021). NGS-based MRD monitoring can further enhance prognostic assessment when applied alongside multiparameter FC, detecting persistent or emerging variants that may indicate molecular persistence or disease evolution (Walter et al., 2021).

### Advancing LSC detection with spectral flow cytometry

Spectral FC enables the simultaneous analysis of 20+ markers in a single tube (Bonilla et al., 2020), offering a powerful tool to interrogate rare leukemic subpopulations, including LSCs, without requiring multiple tubes or complex panel splitting. A recently developed 29-color single-tube spectral assay further exemplifies the potential of this technology (Zhang et al., 2025). Compared with conventional multicolor cytometry, spectral FC minimizes sample consumption and acquisition variability while enabling a more refined “different-from-normal” analysis strategy (Li K. et al., 2024). Dimensionality reduction algorithms further can enhance the resolution of immunophenotypic heterogeneity (Ferrer-Font et al., 2020), with LSCs occupying distinct and reproducible positions separate from normal HSPCs. Building on these advances, recent efforts have shifted from reliance on single surface markers to profiling co-expression patterns of multiple antigens (Haubner et al., 2019). Flow cytometric assessment of co-expression signatures improves diagnostic specificity, overcoming the phenotypic overlap between LSCs and normal HSCs, while also carrying therapeutic implications by informing the design of combinatorial targeted therapies that increase selectivity and minimize off-target toxicity. Collectively, these advances highlight how next-generation cytometry can refine LSC detection and accelerate translation into precision MRD monitoring and personalized therapeutic approaches in AML.

Future optimization of FC-based strategies is expected to further improve AML MRD detection sensitivity and specificity. For example, standardization of antibody panels across institutions, harmonized instrument settings and analysis thresholds, implementation of automated high-dimensional analysis pipelines, and integration with machine learning–based clustering tools will enhance reproducibility and objectivity in rare LSC detection. Increasing total event acquisition is likely to increase sensitivity of MRD detection as well. Finally, aligning FC-based and molecular MRD assessments may provide complementary information on residual disease and treatment response. Further studies are needed to determine whether single or combined MRD approaches offer the most accurate prognostic value and how they should be applied to guide personalized treatment decisions.

## References

[B1] Abacka H. Masoni S. Poli G. Huang P. Gusso F. Granchi C. (2024). SMS121, a new inhibitor of CD36, impairs fatty acid uptake and viability of acute myeloid leukemia. Sci. Rep. 14 (1), 9104. 10.1038/s41598-024-58689-1 38643249 PMC11032350

[B2] Akashi K. (2015). “TIM-3 is a novel therapeutic target for eradicating acute myelogenous leukemia stem cells,” in Nakao, K., Minato, N., Uemoto, S., Editors. Innovative medicine: basic research and development. Tokyo: Springer. 29787181

[B3] Angelini D. F. Ottone T. Guerrera G. Lavorgna S. Cittadini M. Buccisano F. (2015). A leukemia-associated CD34/CD123/CD25/CD99+ immunophenotype identifies FLT3-Mutated clones in acute myeloid leukemia. Clin. Cancer Res. 21 (17), 3977–3985. 10.1158/1078-0432.CCR-14-3186 25957287

[B4] Antony M. L. Chang D. Noble-Orcutt K. E. Kay A. Jensen J. L. Mohei H. (2023). CD69 marks a subpopulation of acute myeloid leukemia with enhanced colony forming capacity and a unique signaling activation state. Leuk. Lymphoma 64 (7), 1262–1274. 10.1080/10428194.2023.2207698 37161853

[B5] Askmyr M. Ågerstam H. Hansen N. Gordon S. Arvanitakis A. Rissler M. (2013). Selective killing of candidate AML stem cells by antibody targeting of IL1RAP. Blood 121 (18), 3709–3713. 10.1182/blood-2012-09-458935 23479569

[B6] Bajaj J. Konuma T. Lytle N. K. Kwon H. Y. Ablack J. N. Cantor J. M. (2016). CD98-Mediated adhesive signaling enables the establishment and propagation of acute myelogenous leukemia. Cancer Cell 30 (5), 792–805. 10.1016/j.ccell.2016.10.003 27908736 PMC5137811

[B7] Baum C. M. Weissman I. L. Tsukamoto A. S. Buckle A. M. Peault B. (1992). Isolation of a candidate human hematopoietic stem-cell population. Proc. Natl. Acad. Sci. U. S. A. 89 (7), 2804–2808. 10.1073/pnas.89.7.2804 1372992 PMC48751

[B8] Biondi M. Cerina B. Tomasoni C. Dotti G. Tettamanti S. Biondi A. (2021). Combining the expression of CD33.CAR and CXCR4 to increase CAR-CIK cell homing to bone marrow niche and leukemic stem cell eradication in acute myeloid leukemia. Blood 138, 2791. 10.1182/blood-2021-152394

[B9] Blachly J. S. Walter R. B. Hourigan C. S. (2022). The present and future of measurable residual disease testing in acute myeloid leukemia. Haematologica 107 (12), 2810–2822. 10.3324/haematol.2022.282034 36453518 PMC9713561

[B10] Blume R. Rempel E. Manta L. Saeed B. R. Wang W. Raffel S. (2018). The molecular signature of AML with increased ALDH activity suggests a stem cell origin. Leuk. Lymphoma 59 (9), 2201–2210. 10.1080/10428194.2017.1422862 29334844

[B11] Boesch M. Cosma A. Sopper S. (2018). Flow cytometry: to dump or not to dump. J. Immunol. 201 (7), 1813–1815. 10.4049/jimmunol.1801037 30224359

[B12] Bonilla D. L. Reinin G. Chua E. (2020). Full spectrum flow cytometry as a powerful technology for cancer immunotherapy research. Front. Mol. Biosci. 7, 612801. 10.3389/fmolb.2020.612801 33585561 PMC7878389

[B13] Bonnet D. Dick J. E. (1997). Human acute myeloid leukemia is organized as a hierarchy that originates from a primitive hematopoietic cell. Nat. Med. 3 (7), 730–737. 10.1038/nm0797-730 9212098

[B14] Boyer T. Gonzales F. Plesa A. Peyrouze P. Barthelemy A. Guihard S. (2018). Flow cytometry to estimate leukemia stem cells in primary acute myeloid leukemia and in patient-derived-xenografts, at diagnosis and follow up. J. Vis. Exp. (133), 56976. 10.3791/56976 29630051 PMC5933244

[B15] Brendel C. Mohr B. Schimmelpfennig C. Müller J. Bornhäuser M. Schmidt M. (1999). Detection of cytogenetic aberrations both in CD90 (Thy-1)-positive and (Thy-1)-negative stem cell (CD34) subfractions of patients with acute and chronic myeloid leukemias. Leukemia 13 (11), 1770–1775. 10.1038/sj.leu.2401543 10557051

[B16] Buccisano F. Rossi F. M. Venditti A. Del Poeta G. Cox M. C. Abbruzzese E. (2004). CD90/Thy‐1 is preferentially expressed on blast cells of high risk acute myeloid leukaemias. Br. Journal Haematology 125 (2), 203–212. 10.1111/j.1365-2141.2004.04883.x 15059143

[B17] Cerny J. Yu H. Ramanathan M. Raffel G. D. Walsh W. V. Fortier N. (2013). Expression of CD25 independently predicts early treatment failure of acute myeloid leukaemia (AML). Br. J. Haematol. 160 (2), 262–266. 10.1111/bjh.12109 23116454

[B18] Charrad R. S. Gadhoum Z. Qi J. Glachant A. Allouche M. Jasmin C. (2002). Effects of anti-CD44 monoclonal antibodies on differentiation and apoptosis of human myeloid leukemia cell lines. Blood 99 (1), 290–299. 10.1182/blood.v99.1.290 11756184

[B19] Cheng Y. Jia M. Chen Y. Zhao H. Luo Z. Tang Y. (2016). Re-evaluation of various molecular targets located on CD34(+)CD38(-)Lin(-) leukemia stem cells and other cell subsets in pediatric acute myeloid leukemia. Oncol. Lett. 11 (1), 891–897. 10.3892/ol.2015.3972 26870301 PMC4726975

[B22] Chung S. S. Eng W. S. Hu W. Khalaj M. Garrett-Bakelman F. E. Tavakkoli M. (2017). CD99 is a therapeutic target on disease stem cells in myeloid malignancies. Sci. Transl. Med. 9 (374), eaaj2025. 10.1126/scitranslmed.aaj2025 28123069 PMC5624309

[B23] Cloos J. Harris J. R. Janssen J. J. W. M. Kelder A. Huang F. Sijm G. (2018). Comprehensive protocol to sample and process bone marrow for measuring measurable residual disease and leukemic stem cells in acute myeloid leukemia. J. Vis. Exp. (133), e56386. 10.3791/56386 29553571 PMC5931431

[B24] Coles S. J. Wang E. C. Y. Man S. Hills R. K. Burnett A. K. Tonks A. (2011). CD200 expression suppresses natural killer cell function and directly inhibits patient anti-tumor response in acute myeloid leukemia. Leukemia 25 (5), 792–799. 10.1038/leu.2011.1 21274000 PMC3093357

[B25] Coles S. J. Hills R. K. Wang E. C. Y. Burnett A. K. Man S. Darley R. L. (2012). Expression of CD200 on AML blasts directly suppresses memory T-cell function. Leukemia 26 (9), 2148–2151. 10.1038/leu.2012.77 22430635 PMC3460216

[B26] Daria D. Kirsten N. Muranyi A. Mulaw M. Ihme S. Kechter A. (2016). GPR56 contributes to the development of acute myeloid leukemia in mice. Leukemia 30 (8), 1734–1741. 10.1038/leu.2016.76 27063597

[B27] Darwish N. H. Sudha T. Godugu K. Elbaz O. Abdelghaffar H. A. Hassan E. E. A. (2016). Acute myeloid leukemia stem cell markers in prognosis and targeted therapy: potential impact of BMI-1, TIM-3 and CLL-1. Oncotarget 7 (36), 57811–57820. 10.18632/oncotarget.11063 27506934 PMC5295391

[B28] Das N. Panda D. Gajendra S. Gupta R. Thakral D. Kaur G. (2024). Immunophenotypic characterization of leukemic stem cells in acute myeloid leukemia using single tube 10-colour panel by multiparametric flow cytometry: deciphering the spectrum, complexity and immunophenotypic heterogeneity. Int. J. Lab. Hematol. 46 (4), 646–656. 10.1111/ijlh.14250 38456256

[B29] Dohner H. Weisdorf D. J. Bloomfield C. D. (2015). Acute myeloid leukemia. N. Engl. J. Med. 373 (12), 1136–1152. 10.1056/NEJMra1406184 26376137

[B30] Dohner H. Wei A. H. Appelbaum F. R. Craddock C. DiNardo C. D. Dombret H. (2022). Diagnosis and management of AML in adults: 2022 recommendations from an international expert panel on behalf of the ELN. Blood 140 (12), 1345–1377. 10.1182/blood.2022016867 35797463

[B31] Du W. Hu Y. Lu C. Li J. Liu W. He Y. (2015). Cluster of differentiation 96 as a leukemia stem cell-specific marker and a factor for prognosis evaluation in leukemia. Mol. Clin. Oncol. 3 (4), 833–838. 10.3892/mco.2015.552 26171191 PMC4487019

[B32] Eppert K. Takenaka K. Lechman E. R. Waldron L. Nilsson B. van Galen P. (2011). Stem cell gene expression programs influence clinical outcome in human leukemia. Nat. Med. 17 (9), 1086–1093. 10.1038/nm.2415 21873988

[B33] Farge T. Nakhle J. Lagarde D. Cognet G. Polley N. Castellano R. (2023). CD36 drives metastasis and relapse in acute myeloid leukemia. Cancer Res. 83 (17), 2824–2838. 10.1158/0008-5472.CAN-22-3682 37327406 PMC10472106

[B34] Ferrer-Font L. Mayer J. U. Old S. Hermans I. F. Irish J. Price K. M. (2020). High-dimensional data analysis algorithms yield comparable results for mass cytometry and spectral flow cytometry data. Cytom. A 97 (8), 824–831. 10.1002/cyto.a.24016 32293794 PMC7682594

[B35] Feuring-Buske M. Hogge D. E. (2001). Hoechst 33342 efflux identifies a subpopulation of cytogenetically normal CD34(+)CD38(-) progenitor cells from patients with acute myeloid leukemia. Blood 97 (12), 3882–3889. 10.1182/blood.v97.12.3882 11389030

[B36] Flores-Montero J. Sanoja-Flores L. Paiva B. Puig N. García-Sánchez O. Böttcher S. (2017). Next generation flow for highly sensitive and standardized detection of minimal residual disease in multiple myeloma. Leukemia 31 (10), 2094–2103. 10.1038/leu.2017.29 28104919 PMC5629369

[B37] Freeman S. D. Hills R. K. Virgo P. Khan N. Couzens S. Dillon R. (2018). Measurable residual disease at induction redefines partial response in acute myeloid leukemia and stratifies outcomes in patients at standard risk without NPM1 mutations. J. Clin. Oncol. 36 (15), 1486–1497. 10.1200/JCO.2017.76.3425 29601212 PMC5959196

[B38] Goardon N. Marchi E. Atzberger A. Quek L. Schuh A. Soneji S. (2011). Coexistence of LMPP-Like and GMP-Like leukemia stem cells in acute myeloid leukemia. Cancer Cell 19 (1), 138–152. 10.1016/j.ccr.2010.12.012 21251617

[B39] Grenier J. M. P. Testut C. Fauriat C. Mancini S. J. C. Aurrand-Lions M. (2021). Adhesion molecules involved in stem cell niche retention during normal haematopoiesis and in acute myeloid leukaemia. Front. Immunol. 12, 756231. 10.3389/fimmu.2021.756231 34867994 PMC8636127

[B40] Guerrero-Rodriguez S. L. Mata-Cruz C. Pérez-Tapia S. M. Velasco-Velázquez M. A. (2022). Role of CD36 in cancer progression, stemness, and targeting. Front. Cell Dev. Biol. 10, 1079076. 10.3389/fcell.2022.1079076 36568966 PMC9772993

[B41] Gutjahr J. C. Bayer E. Yu X. Laufer J. M. Höpner J. P. Tesanovic S. (2021). CD44 engagement enhances acute myeloid leukemia cell adhesion to the bone marrow microenvironment by increasing VLA-4 avidity. Haematologica 106 (8), 2102–2113. 10.3324/haematol.2019.231944 32616529 PMC8327716

[B42] Guy J. Antony-Debré I. Benayoun E. Arnoux I. Fossat C. Le Garff-Tavernier M. (2013). Flow cytometry thresholds of myeloperoxidase detection to discriminate between acute lymphoblastic or myeloblastic leukaemia. Br. J. Haematol. 161 (4), 551–555. 10.1111/bjh.12277 23432206

[B43] Hanekamp D. Denys B. Kaspers G. J. L. Te Marvelde J. G. Schuurhuis G. J. De Haas V. (2018). Leukaemic stem cell load at diagnosis predicts the development of relapse in young acute myeloid leukaemia patients. Br. J. Haematol. 183 (3), 512–516. 10.1111/bjh.14991 29076143

[B44] Hanekamp D. Bachas C. van de Loosdrecht A. Ossenkoppele G. Cloos J. (2020). Re: myeloblasts in normal bone marrows expressing leukaemia-associated immunophenotypes. Pathology 52 (2), 289–291. 10.1016/j.pathol.2019.09.021 31883670

[B45] Haubner S. Perna F. Köhnke T. Schmidt C. Berman S. Augsberger C. (2019). Coexpression profile of leukemic stem cell markers for combinatorial targeted therapy in AML. Leukemia 33 (1), 64–74. 10.1038/s41375-018-0180-3 29946192 PMC6326956

[B46] Hayes G. M. Chinn L. Cantor J. M. Cairns B. Levashova Z. Tran H. (2015). Antitumor activity of an anti-CD98 antibody. Int. J. Cancer 137 (3), 710–720. 10.1002/ijc.29415 25556716 PMC6680144

[B47] Heo S. K. Noh E. K. Ju L. J. Sung J. Y. Jeong Y. K. Cheon J. (2020). CD45(dim)CD34(+)CD38(-)CD133(+) cells have the potential as leukemic stem cells in acute myeloid leukemia. BMC Cancer 20 (1), 285. 10.1186/s12885-020-06760-1 32252668 PMC7137473

[B48] Herrmann H. Sadovnik I. Eisenwort G. Rülicke T. Blatt K. Herndlhofer S. (2020). Delineation of target expression profiles in CD34+/CD38-and CD34+/CD38+ stem and progenitor cells in AML and CML. Blood Adv. 4 (20), 5118–5132. 10.1182/bloodadvances.2020001742 33085758 PMC7594398

[B49] Heuser M. Freeman S. D. Ossenkoppele G. J. Buccisano F. Hourigan C. S. Ngai L. L. (2021). 2021 update on MRD in acute myeloid leukemia: a consensus document from the european LeukemiaNet MRD working party. Blood, J. Am. Soc. Hematol. 138 (26), 2753–2767. 10.1182/blood.2021013626 34724563 PMC8718623

[B50] Hinterbrandner M. Kallen N. M. Lüthi U. Pabst T. Van Rompaey L. Leupin N. (2017). Blocking CD70/CD27 signaling in combination with hypomethylating agents eradicates human CD34 AML stem and progenitor cells *in vitro* and *in vivo* . Blood 130, 2652. 10.1182/BLOOD.V130.SUPPL_1.2652.2652

[B51] Ho T. C. LaMere M. Stevens B. M. Ashton J. M. Myers J. R. O'Dwyer K. M. (2016). Evolution of acute myelogenous leukemia stem cell properties after treatment and progression. Blood 128 (13), 1671–1678. 10.1182/blood-2016-02-695312 27421961 PMC5043124

[B52] Ho J. M. Dobson S. M. Voisin V. McLeod J. Kennedy J. A. Mitchell A. (2020). CD200 expression marks leukemia stem cells in human AML. Blood Adv. 4 (21), 5402–5413. 10.1182/bloodadvances.2020001802 33147339 PMC7656934

[B53] Hoang V. T. Buss E. C. Wang W. Hoffmann I. Raffel S. Zepeda-Moreno A. (2015). The rarity of ALDH(+) cells is the key to separation of normal *versus* leukemia stem cells by ALDH activity in AML patients. Int. J. Cancer 137 (3), 525–536. 10.1002/ijc.29410 25545165 PMC4755039

[B54] Holden J. T. Geller R. B. Farhi D. C. Holland H. K. Stempora L. L. Phillips C. N. (1995). Characterization of Thy-1 (CDw90) expression in CD34+ acute leukemia. Blood 86 (1), 60–65. 10.1182/blood.v86.1.60.bloodjournal86160 7540889

[B55] Hosen N. Park C. Y. Tatsumi N. Oji Y. Sugiyama H. Gramatzki M. (2007). CD96 is a leukemic stem cell-specific marker in human acute myeloid leukemia. Proc. Natl. Acad. Sci. U. S. A. 104 (26), 11008–11013. 10.1073/pnas.0704271104 17576927 PMC1904175

[B56] Hwang K. Park C. J. Jang S. Chi H. S. Kim D. Y. Lee J. H. (2012). Flow cytometric quantification and immunophenotyping of leukemic stem cells in acute myeloid leukemia. Ann. Hematol. 91 (10), 1541–1546. 10.1007/s00277-012-1501-7 22669506

[B57] Inaba T. Shimazaki C. Sumikuma T. Takahashi R. Hirai H. Kikuta T. (1997). Flow cytometric analysis of Thy-1 expression in CD34-positive acute leukemia. Int. J. Hematol. 66 (3), 315–323. 10.1016/s0925-5710(97)00051-0 9401277

[B58] Ivey A. Hills R. K. Simpson M. A. Jovanovic J. V. Gilkes A. Grech A. (2016). Assessment of minimal residual disease in standard-risk AML. N. Engl. J. Med. 374 (5), 422–433. 10.1056/NEJMoa1507471 26789727

[B59] Iwasaki M. Liedtke M. Gentles A. J. Cleary M. L. (2015). CD93 marks a non-quiescent human leukemia stem cell population and is required for development of MLL-rearranged acute myeloid leukemia. Cell Stem Cell 17 (4), 412–421. 10.1016/j.stem.2015.08.008 26387756 PMC4592469

[B60] Jan M. Chao M. P. Cha A. C. Alizadeh A. A. Gentles A. J. Weissman I. L. (2011). Prospective separation of normal and leukemic stem cells based on differential expression of TIM3, a human acute myeloid leukemia stem cell marker. Proc. Natl. Acad. Sci. U. S. A. 108 (12), 5009–5014. 10.1073/pnas.1100551108 21383193 PMC3064328

[B61] Jan M. Snyder T. M. Corces-Zimmerman M. R. Vyas P. Weissman I. L. Quake S. R. (2012). Clonal evolution of preleukemic hematopoietic stem cells precedes human acute myeloid leukemia. Sci. Transl. Med. 4 (149), 149ra118. 10.1126/scitranslmed.3004315 22932223 PMC4045621

[B62] Jia J. Liu B. Wang D. Wang X. Song L. Ren Y. (2022). CD93 promotes acute myeloid leukemia development and is a potential therapeutic target. Exp. Cell Res. 420 (2), 113361. 10.1016/j.yexcr.2022.113361 36152731

[B63] Jin L. Hope K. J. Zhai Q. Smadja-Joffe F. Dick J. E. (2006). Targeting of CD44 eradicates human acute myeloid leukemic stem cells. Nat. Med. 12 (10), 1167–1174. 10.1038/nm1483 16998484

[B64] Jin L. Lee E. M. Ramshaw H. S. Busfield S. J. Peoppl A. G. Wilkinson L. (2009). Monoclonal antibody-mediated targeting of CD123, IL-3 receptor alpha chain, eliminates human acute myeloid leukemic stem cells. Cell Stem Cell 5 (1), 31–42. 10.1016/j.stem.2009.04.018 19570512

[B65] Jordan C. T. Upchurch D. Szilvassy S. J. Guzman M. L. Howard D. S. Pettigrew A. L. (2000). The interleukin-3 receptor alpha chain is a unique marker for human acute myelogenous leukemia stem cells. Leukemia 14 (10), 1777–1784. 10.1038/sj.leu.2401903 11021753

[B66] Joshi K. Zhang L. Breslin S J P. Zhang J. (2019). Leukemia stem cells in the pathogenesis, progression, and treatment of acute myeloid leukemia. Adv. Exp. Med. Biol. 1143, 95–128. 10.1007/978-981-13-7342-8_5 31338817 PMC9056110

[B67] Joudinaud R. Boyer T. (2021). Stem cells in myelodysplastic syndromes and acute myeloid leukemia: first cousins or unrelated entities? Front. Oncol. 11, 730899. 10.3389/fonc.2021.730899 34490124 PMC8417738

[B68] Kageyama Y. Miwa H. Arakawa R. Tawara I. Ohishi K. Masuya M. (2018). Expression of CD25 fluctuates in the leukemia-initiating cell population of CD25-positive AML. PLoS One 13 (12), e0209295. 10.1371/journal.pone.0209295 30550585 PMC6294374

[B69] Kersten B. Valkering M. Wouters R. van Amerongen R. Hanekamp D. Kwidama Z. (2016). CD45RA, a specific marker for leukaemia stem cell sub-populations in acute myeloid leukaemia. Br. J. Haematol. 173 (2), 219–235. 10.1111/bjh.13941 26814163

[B70] Kikushige Y. Miyamoto T. Yuda J. Jabbarzadeh-Tabrizi S. Shima T. Takayanagi S. i. (2015). A TIM-3/Gal-9 autocrine stimulatory loop drives self-renewal of human myeloid leukemia stem cells and leukemic progression. Cell Stem Cell 17 (3), 341–352. 10.1016/j.stem.2015.07.011 26279267

[B71] Kinstrie R. Horne G. A. Morrison H. Irvine D. Munje C. Castañeda E. G. (2020). CD93 is expressed on chronic myeloid leukemia stem cells and identifies a quiescent population which persists after tyrosine kinase inhibitor therapy. Leukemia 34 (6), 1613–1625. 10.1038/s41375-019-0684-5 31896780 PMC7272220

[B72] Kozii R. Wilson J. Persichetti J. Phelps V. Ball S. E. Ball E. D. (1997). Thy-1 expression on blast cells from adult patients with acute myeloid leukemia. Leuk. Res. 21 (5), 381–385. 10.1016/s0145-2126(96)00081-1 9225063

[B73] Landberg N. von Palffy S. Askmyr M. Lilljebjörn H. Sandén C. Rissler M. (2018). CD36 defines primitive chronic myeloid leukemia cells less responsive to imatinib but vulnerable to antibody-based therapeutic targeting. Haematologica 103 (3), 447–455. 10.3324/haematol.2017.169946 29284680 PMC5830390

[B74] Lapidot T. Sirard C. Vormoor J. Murdoch B. Hoang T. Caceres-Cortes J. (1994). A cell initiating human acute myeloid leukaemia after transplantation into SCID mice. Nature 367 (6464), 645–648. 10.1038/367645a0 7509044

[B75] Larsen H. O. Roug A. S. Just T. Brown G. D. Hokland P. (2012). Expression of the hMICL in acute myeloid leukemia-a highly reliable disease marker at diagnosis and during follow-up. Cytom. B Clin. Cytom. 82 (1), 3–8. 10.1002/cyto.b.20614 22173921

[B76] Li S. Q. Xu L. P. Wang Y. Zhang X. H. Chen H. Chen Y. H. (2022). An LSC-Based MRD assay to complement the traditional MFC method for prediction of AML relapse: a prospective study. Blood 140 (5), 516–520. 10.1182/blood.2021014604 35613411

[B77] Li H. Cao Z. Liu Y. Xue Z. Li Y. Xing H. (2024a). Slow-replicating leukemia cells represent a leukemia stem cell population with high cell-surface CD74 expression. Mol. Oncol. 18 (10), 2554–2568. 10.1002/1878-0261.13690 38922758 PMC11459046

[B78] Li K. Chen M. Gong M. Lu P. Wang H. (2024b). A novel full-spectral flow cytometric assay for detection of measurable residual disease in patients with B-Lymphoblastic leukemia. Blood 144 (Suppl. 1), 5911–5912. 10.1182/blood-2024-203516

[B79] Li S. Q. Xu L. P. Wang Y. Zhang X. H. Chen H. Chen Y. H. (2025). Leukemia stem cells for relapse prediction in AML patients receiving allografts: long-term follow-up of a prospective study. Bone Marrow Transpl. 60, 1472–1479. 10.1038/s41409-025-02699-8 40849363

[B80] Liu Y. Wang G. Zhang J. Chen X. Xu H. Heng G. (2021). CD9, a potential leukemia stem cell marker, regulates drug resistance and leukemia development in acute myeloid leukemia. Stem Cell Res. Ther. 12 (1), 86. 10.1186/s13287-021-02155-6 33494824 PMC7836575

[B81] Loghavi S. DiNardo C. D. Furudate K. Takahashi K. Tanaka T. Short N. J. (2021). Flow cytometric immunophenotypic alterations of persistent clonal haematopoiesis in remission bone marrows of patients with NPM1-mutated acute myeloid leukaemia. Br. J. Haematol. 192 (6), 1054–1063. 10.1111/bjh.17347 33618432 PMC8944196

[B82] Maganti H. Visram A. Shorr R. Fulcher J. Sabloff M. Allan D. S. (2020). Plerixafor in combination with chemotherapy And/or hematopoietic cell transplantation to treat acute leukemia: a systematic review and metanalysis of preclinical and clinical studies. Leuk. Res. 97, 106442. 10.1016/j.leukres.2020.106442 32877869

[B83] Majeti R. Park C. Y. Weissman I. L. (2007). Identification of a hierarchy of multipotent hematopoietic progenitors in human cord blood. Cell Stem Cell 1 (6), 635–645. 10.1016/j.stem.2007.10.001 18371405 PMC2292126

[B84] Majeti R. Chao M. P. Alizadeh A. A. Pang W. W. Weissman I. L. (2008). CD47 is an independent prognostic factor and therapeutic antibody target on human acute myeloid leukemia stem cells. Blood 112 (11), 766–284. 10.1182/blood.v112.11.766.766 PMC2726837 19632179

[B85] Majeti R. Chao M. P. Alizadeh A. A. Pang W. W. Jaiswal S. Gibbs K. D. Jr (2009). CD47 is an adverse prognostic factor and therapeutic antibody target on human acute myeloid leukemia stem cells. Cell 138 (2), 286–299. 10.1016/j.cell.2009.05.045 19632179 PMC2726837

[B86] Marques-Piubelli M. L. Kumar B. Basar R. Panowski S. Srinivasan S. Norwood K. (2024). Increased expression of CD70 in relapsed acute myeloid leukemia after hypomethylating agents. Virchows Arch. 485 (5), 937–941. 10.1007/s00428-024-03741-8 38388965 PMC11564407

[B87] Martin G. H. Desrichard A. Chung S. S. Woolthuis C. Hu W. Garrett-Bakelman F. E. (2016). CD97 is a critical regulator of acute myeloid leukemia stem cell function. Blood 128 (22), 1077. 10.1182/blood.v128.22.1077.1077 PMC6781010 31371381

[B88] Martin G. H. Roy N. Chakraborty S. Desrichard A. Chung S. S. Woolthuis C. M. (2019). CD97 is a critical regulator of acute myeloid leukemia stem cell function. J. Exp. Med. 216 (10), 2362–2377. 10.1084/jem.20190598 31371381 PMC6781010

[B89] Medina-Herrera A. Sarasquete M. E. Jiménez C. Puig N. García-Sanz R. (2023). Minimal residual disease in multiple myeloma: past, present, and future. Cancers (Basel) 15 (14), 3687. 10.3390/cancers15143687 37509348 PMC10377959

[B90] Menssen A. J. Hudson C. A. Alonzo T. Gerbing R. Pardo L. Leonti A. (2024). CD74 is expressed in a subset of pediatric acute myeloid leukemia patients and is a promising target for therapy: a report from the children's oncology group. Haematologica 109 (10), 3182–3193. 10.3324/haematol.2023.283757 38299667 PMC11443400

[B91] Metois A. Bordeleau M. E. Theret L. Hajmirza A. Moujaber O. Spinella J. F. (2025). IL1RAP is an immunotherapeutic target for normal karyotype triple-mutated acute myeloid leukemia. Biomark. Res. 13 (1), 61. 10.1186/s40364-025-00769-z 40229904 PMC11995633

[B92] Moshaver B. van Rhenen A. Kelder A. van der Pol M. Terwijn M. Bachas C. (2008). Identification of a small subpopulation of candidate leukemia-initiating cells in the side population of patients with acute myeloid leukemia. Stem Cells 26 (12), 3059–3067. 10.1634/stemcells.2007-0861 19096043

[B93] Moshaver B. Wouters R. F. Kelder A. Ossenkoppele G. J. Westra G. A. H. Kwidama Z. (2019). Relationship between CD34/CD38 and side population (SP) defined leukemia stem cell compartments in acute myeloid leukemia. Leuk. Res. 81, 27–34. 10.1016/j.leukres.2019.04.004 31002948

[B94] National Cancer Institute (2024). Cancer stat facts: Acute myeloid leukemia (AML). Available online at: https://seer.cancer.gov/statfacts/html/amyl.html (Accessed May 05, 2025).

[B95] Ng S. W. Mitchell A. Kennedy J. A. Chen W. C. McLeod J. Ibrahimova N. (2016). A 17-gene stemness score for rapid determination of risk in acute leukaemia. Nature 540 (7633), 433–437. 10.1038/nature20598 27926740

[B96] Ngai L. L. Hanekamp D. Janssen F. Carbaat-Ham J. Hofland M. A. M. A. Fayed M. M. H. E. (2023). Prospective validation of the prognostic relevance of CD34+CD38- AML stem cell frequency in the HOVON-SAKK132 trial. Blood 141 (21), 2657–2661. 10.1182/blood.2022019160 36898087 PMC10646801

[B97] Ngai L. L. Reuvekamp T. Hanekamp D. Janssen F. Marsbergen L. O. v. Carbaat-Ham J. (2025). Different features of acute myeloid leukemia stem cell quantification in intensively treated patients. Haematologica. 10.3324/haematol.2024.287090 40304060 PMC12666270

[B99] Noordhuis P. Terwijn M. Rutten A. P. Smit L. Ossenkoppele G. J. Schuurhuis G. J. (2010). Targeting of CLEC12A in acute myeloid leukemia by antibody-drug-conjugates and bispecific CLL-1×CD3 BiTE antibody. Blood 116 (21), 2890. 10.1182/blood.v116.21.2890.2890

[B100] Notta F. Doulatov S. Laurenti E. Poeppl A. Jurisica I. Dick J. E. (2011). Isolation of single human hematopoietic stem cells capable of long-term multilineage engraftment. Science 333 (6039), 218–221. 10.1126/science.1201219 21737740

[B101] Ochsenbein A. F. Pabst T. Höpner S. Bacher V. U. Hinterbrandner M. Banz Y. (2019). Targeting CD70 with cusatuzumab eliminates acute myeloid leukemia stem cells in humans. Blood 134, 234. 10.1182/blood-2019-129916 32601337

[B102] Ogana H. A. Hurwitz S. Wei N. Lee E. Morris K. Parikh K. (2024). Targeting integrins in drug-resistant acute myeloid leukaemia. Br. J. Pharmacol. 181 (2), 295–316. 10.1111/bph.16149 37258706

[B103] Pabst C. Bergeron A. Lavallée V. P. Yeh J. Gendron P. Norddahl G. L. (2016). GPR56 identifies primary human acute myeloid leukemia cells with high repopulating potential *in vivo* . Blood 127 (16), 2018–2027. 10.1182/blood-2015-11-683649 26834243

[B104] Pei S. Shelton I. T. Gillen A. E. Stevens B. M. Gasparetto M. Wang Y. (2023). A novel type of monocytic leukemia stem cell revealed by the clinical use of Venetoclax-Based therapy. Cancer Discov. 13 (9), 2032–2049. 10.1158/2159-8290.CD-22-1297 37358260 PMC10527971

[B105] Petersen M. A. Bill M. Rosenberg C. A. (2021). OMIP 072: a 15-color panel for immunophenotypic identification, quantification, and characterization of leukemic stem cells in children with acute myeloid leukemia. Cytom. A 99 (4), 382–387. 10.1002/cyto.a.24284 33369057

[B106] Qiu G. Xu X. Liu Q. F. (2022). CD69 marks leukemic regenerating cells and regulates their metabolism in AML. Blood 140 (Suppl. 1), 8780–8782. 10.1182/blood-2022-168383

[B107] Quek L. Otto G. Garnett C. Lhermitte L. Lau I-J. Karamitros D. (2014). Functional and genetic heterogeneity of distinct leukemic stem cell populations in CD34- human acute myeloid leukemia. Blood 124 (21), 15. 10.1182/blood.V124.21.15.15

[B108] Quek L. Otto G. W. Garnett C. Lhermitte L. Karamitros D. Stoilova B. (2016). Genetically distinct leukemic stem cells in human CD34 acute myeloid leukemia are arrested at a hemopoietic precursor-like stage. J. Exp. Med. 213 (8), 1513–1535. 10.1084/jem.20151775 27377587 PMC4986529

[B109] Ran D. Schubert M. Pietsch L. Taubert I. Wuchter P. Eckstein V. (2009). Aldehyde dehydrogenase activity among primary leukemia cells is associated with stem cell features and correlates with adverse clinical outcomes. Exp. Hematol. 37 (12), 1423–1434. 10.1016/j.exphem.2009.10.001 19819294

[B110] Reinisch A. Majeti R. (2016). Sticking it to the niche: CD98 mediates critical adhesive signals in AML. Cancer Cell 30 (5), 662–664. 10.1016/j.ccell.2016.10.014 27846387

[B111] Reuvekamp T. Janssen L. L. G. Ngai L. L. Carbaat-Ham J. den Hartog D. Scholten W. J. (2025a). The role of the primitive marker CD133 in CD34-negative acute myeloid leukemia for the detection of leukemia stem cells. Cytom. B Clin. Cytom. 108 (1), 23–34. 10.1002/cyto.b.22201 39177948

[B112] Reuvekamp T. Ngai L. L. den Hartog D. Carbaat-Ham J. Fayed M. M. H. E. Scholten W. J. (2025b). CD34+CD38-leukemia stem cells predict clinical outcomes in acute myeloid leukemia patients treated non-intensively with hypomethylating agents. Leukemia 39 (4), 972–975. 10.1038/s41375-025-02539-0 40016301 PMC11976276

[B113] Richards R. M. Zhao F. Freitas K. A. Parker K. R. Xu P. Fan A. (2021). NOT-gated CD93 CAR T cells effectively target AML with minimized endothelial cross-reactivity. Blood Cancer Discov. 2 (6), 648–665. 10.1158/2643-3230.BCD-20-0208 34778803 PMC8580619

[B114] Riether C. Schürch C. M. Bührer E. D. Hinterbrandner M. Huguenin A. L. Hoepner S. (2017). CD70/CD27 signaling promotes blast stemness and is a viable therapeutic target in acute myeloid leukemia. J. Exp. Med. 214 (2), 359–380. 10.1084/jem.20152008 28031480 PMC5294846

[B115] Roboz G. J. Ritchie E. K. Dault Y. Lam L. Marshall D. C. Cruz N. M. (2018). Phase I trial of plerixafor combined with decitabine in newly diagnosed older patients with acute myeloid leukemia. Haematologica 103 (8), 1308–1316. 10.3324/haematol.2017.183418 29724902 PMC6068018

[B116] Saito Y. Morishita K. (2015). Maintenance of leukemic and normal hematopoietic stem cells in bone marrow niches by EVI1-regulated GPR56. Rinsho Ketsueki 56 (4), 375–383. 10.11406/rinketsu.56.375 25971267

[B117] Saito Y. Kitamura H. Hijikata A. Tomizawa-Murasawa M. Tanaka S. Takagi S. (2010). Identification of therapeutic targets for quiescent, chemotherapy-resistant human leukemia stem cells. Sci. Transl. Med. 2 (17), 17ra9. 10.1126/scitranslmed.3000349 20371479 PMC3005290

[B118] Sallman D. A. Asch A. S. Al Malki M. M. Lee D. J. Donnellan W. B. Marcucci G. (2019). The first-in-class Anti-CD47 antibody magrolimab (5F9) in combination with azacitidine is effective in MDS and AML patients: ongoing phase 1b results. Blood 134, 569. 10.1182/blood-2019-126271

[B119] Sarry J. E. Murphy K. Perry R. Sanchez P. V. Secreto A. Keefer C. (2011). Human acute myelogenous leukemia stem cells are rare and heterogeneous when assayed in NOD/SCID/IL2Rγc-deficient mice. J. Clin. Invest 121 (1), 384–395. 10.1172/JCI41495 21157036 PMC3007135

[B120] Schuurhuis G. J. Meel M. H. Wouters F. Min L. A. Terwijn M. de Jonge N. A. (2013). Normal hematopoietic stem cells within the AML bone marrow have a distinct and higher ALDH activity level than co-existing leukemic stem cells. PLoS One 8 (11), e78897. 10.1371/journal.pone.0078897 24244383 PMC3823975

[B121] Schuurhuis G. J. Heuser M. Freeman S. Béné M. C. Buccisano F. Cloos J. (2018). Minimal/Measurable residual disease in AML: a consensus document from the european LeukemiaNet MRD working party. Blood 131 (12), 1275–1291. 10.1182/blood-2017-09-801498 29330221 PMC5865231

[B122] Shimura-Nukina A. Masamoto Y. Kagoya Y. Arai S. Kurokawa M. (2016). Single-cell gene expression analysis identifies alcam as a novel candidate therapeutic target in AML. Blood 128 (22), 3915. 10.1182/blood.v128.22.3915.3915

[B123] Short N. J. Zhou S. Fu C. Berry D. A. Walter R. B. Freeman S. D. (2020). Association of measurable residual disease with survival outcomes in patients with acute myeloid leukemia: a systematic review and meta-analysis. JAMA Oncol. 6 (12), 1890–1899. 10.1001/jamaoncol.2020.4600 33030517 PMC7545346

[B124] Srinivasan Rajsri K. Roy N. Chakraborty S. (2023). Acute myeloid leukemia stem cells in minimal/measurable residual disease detection. Cancers (Basel) 15 (10), 2866. 10.3390/cancers15102866 37345204 PMC10216329

[B125] Taussig D. C. Vargaftig J. Miraki-Moud F. Griessinger E. Sharrock K. Luke T. (2010). Leukemia-initiating cells from some acute myeloid leukemia patients with mutated nucleophosmin reside in the CD34(-) fraction. Blood 115 (10), 1976–1984. 10.1182/blood-2009-02-206565 20053758 PMC2837317

[B126] Terstappen L. W. Huang S. Safford M. Lansdorp P. M. Loken M. R. (1991). Sequential generations of hematopoietic colonies derived from single nonlineage-committed CD34+CD38-progenitor cells. Blood 77 (6), 1218–1227. 10.1182/blood.v77.6.1218.bloodjournal7761218 1705833

[B127] Terwijn M. Rutten A. P. Kelder A. Snel A. N. Scholten W. J. Zweegman S. (2010). Accurate detection of residual leukemic stem cells in remission bone marrow predicts relapse in acute myeloid leukemia patients. Blood 116 (21), 759–334. 10.1182/blood.v116.21.759.759 20472833

[B128] Terwijn M. van Putten W. L. J. Kelder A. van der Velden V. H. J. Brooimans R. A. Pabst T. (2013). High prognostic impact of flow cytometric minimal residual disease detection in acute myeloid leukemia: data from the HOVON/SAKK AML 42A study. J. Clin. Oncol. 31 (31), 3889–3897. 10.1200/JCO.2012.45.9628 24062400

[B129] Terwijn M. Zeijlemaker W. Kelder A. Rutten A. P. Snel A. N. Scholten W. J. (2014). Leukemic stem cell frequency: a strong biomarker for clinical outcome in acute myeloid leukemia. PLoS One 9 (9), e107587. 10.1371/journal.pone.0107587 25244440 PMC4171508

[B130] Thakral D. Gupta R. Khan A. (2022). Leukemic stem cell signatures in acute myeloid leukemia-targeting the guardians with novel approaches. Stem Cell Rev. Rep. 18 (5), 1756–1773. 10.1007/s12015-022-10349-5 35412219

[B131] Theunissen P. Mejstrikova E. Sedek L. van der Sluijs-Gelling A. J. Gaipa G. Bartels M. (2017). Standardized flow cytometry for highly sensitive MRD measurements in B-cell acute lymphoblastic leukemia. Blood 129 (3), 347–357. 10.1182/blood-2016-07-726307 27903527 PMC5291958

[B132] Touzet L. Dumezy F. Roumier C. Berthon C. Bories C. Quesnel B. (2019). CD9 in acute myeloid leukemia: prognostic role and usefulness to target leukemic stem cells. Cancer Med. 8 (3), 1279–1288. 10.1002/cam4.2007 30740913 PMC6434215

[B133] Unglaub J. M. Brinkmann B. Chen Q. Sedloev D. Bruch P. M. Kolb C. (2023). Improving anti-tumor efficacy of CAR T-Cell therapy for acute myeloid leukemia (AML): results from a multi-drug interaction screening approach. Blood 142, 5969. 10.1182/blood-2023-181251

[B134] van der Linde R. Gatt P. N. Smith S. Fernandez M. A. Vaughan L. Blyth E. (2023). Measurable residual disease (MRD) by flow cytometry in adult B-Acute lymphoblastic leukaemia (B-ALL) and acute myeloid leukaemia (AML): correlation with molecular MRD testing and clinical outcome at one year. Cancers (Basel) 15 (20), 5064. 10.3390/cancers15205064 37894431 PMC10605425

[B135] van der Pol M. A. Feller N. Roseboom M. Moshaver B. Westra G. Broxterman H. J. (2003). Assessment of the normal or leukemic nature of CD34^+^ cells in acute myeloid leukemia with low percentages of CD34 cells. Haematologica 88 (9), 983–993. 12969806

[B136] van Rhenen A. Feller N. Kelder A. Westra A. H. Rombouts E. Zweegman S. (2005). High stem cell frequency in acute myeloid leukemia at diagnosis predicts high minimal residual disease and poor survival. Clin. Cancer Res. 11 (18), 6520–6527. 10.1158/1078-0432.CCR-05-0468 16166428

[B137] van Rhenen A. Moshaver B. Kelder A. Feller N. Nieuwint A. W. M. Zweegman S. (2007a). Aberrant marker expression patterns on the CD34+CD38-stem cell compartment in acute myeloid leukemia allows to distinguish the malignant from the normal stem cell compartment both at diagnosis and in remission. Leukemia 21 (8), 1700–1707. 10.1038/sj.leu.2404754 17525725

[B138] van Rhenen A. van Dongen G. A. M. S. Kelder A. Rombouts E. J. Feller N. Moshaver B. (2007b). The novel AML stem cell associated antigen CLL-1 aids in discrimination between normal and leukemic stem cells. Blood 110 (7), 2659–2666. 10.1182/blood-2007-03-083048 17609428

[B139] Verbeek M. W. C. van der Velden V. H. J. (2024). The evolving landscape of flowcytometric minimal residual disease monitoring in B-Cell precursor acute lymphoblastic leukemia. Int. J. Mol. Sci. 25 (9), 4881. 10.3390/ijms25094881 38732101 PMC11084622

[B140] Vergez F. Green A. S. Tamburini J. Sarry J. E. Gaillard B. Cornillet-Lefebvre P. (2011). High levels of CD34+CD38low/-CD123+ blasts are predictive of an adverse outcome in acute myeloid leukemia: a Groupe Ouest-Est des Leucemies Aigues et Maladies du Sang (GOELAMS) study. Haematologica 96 (12), 1792–1798. 10.3324/haematol.2011.047894 21933861 PMC3232261

[B141] Wagner S. Vadakekolathu J. Tasian S. K. Altmann H. Bornhäuser M. Pockley A. G. (2019). A parsimonious 3-gene signature predicts clinical outcomes in an acute myeloid leukemia multicohort study. Blood Adv. 3 (8), 1330–1346. 10.1182/bloodadvances.2018030726 31015209 PMC6482359

[B142] Walter R. B. Ofran Y. Wierzbowska A. Ravandi F. Hourigan C. S. Ngai L. L. (2021). Measurable residual disease as a biomarker in acute myeloid leukemia: theoretical and practical considerations. Leukemia 35 (6), 1529–1538. 10.1038/s41375-021-01230-4 33758317

[B143] Xie D. Wang B. Bai X. Jin X. Lu W. Zhang M. (2025). Chimeric antigen receptor T cells targeting CD312 as a therapy for acute myeloid leukemia. Chin. Med. J. Engl. 138 (1), 114–116. 10.1097/CM9.0000000000003362 39497369 PMC11717521

[B144] Yabushita T. Satake H. Maruoka H. Morita M. Katoh D. Shimomura Y. (2018). Expression of multiple leukemic stem cell markers is associated with poor prognosis in *de novo* acute myeloid leukemia. Leuk. Lymphoma 59 (9), 2144–2151. 10.1080/10428194.2017.1410888 29251166

[B145] Zeijlemaker W. Kelder A. Wouters R. Valk P. J. M. Witte B. I. Cloos J. (2015). Absence of leukaemic CD34(+) cells in acute myeloid leukaemia is of high prognostic value: a longstanding controversy deciphered. Br. J. Haematol. 171 (2), 227–238. 10.1111/bjh.13572 26104974

[B146] Zeijlemaker W. Kelder A. Oussoren-Brockhoff Y. J. M. Scholten W. J. Snel A. N. Veldhuizen D. (2016). A simple one-tube assay for immunophenotypical quantification of leukemic stem cells in acute myeloid leukemia. Leukemia 30 (2), 439–446. 10.1038/leu.2015.252 26437777

[B147] Zeijlemaker W. Grob T. Meijer R. Hanekamp D. Kelder A. Carbaat-Ham J. C. (2019a). CD34(+)CD38(-) leukemic stem cell frequency to predict outcome in acute myeloid leukemia. Leukemia 33 (5), 1102–1112. 10.1038/s41375-018-0326-3 30542144

[B148] Zeijlemaker W. Kelder A. Cloos J. Schuurhuis G. J. (2019b). Immunophenotypic detection of measurable residual (stem cell) disease using LAIP approach in acute myeloid leukemia. Curr. Protoc. Cytom. 91 (1), e66. 10.1002/cpcy.66 31763792 PMC6856793

[B149] Zhang T. Yang J. Vaikari V. P. Beckford J. S. Wu S. Akhtari M. (2020). Apolipoprotein C2 - CD36 promotes leukemia growth and presents a targetable axis in acute myeloid leukemia. Blood Cancer Discov. 1 (2), 198–213. 10.1158/2643-3230.BCD-19-0077 32944714 PMC7494214

[B150] Zhang Y. Jiang S. He F. Tian Y. Hu H. Gao L. (2023). Single-cell transcriptomics reveals multiple chemoresistant properties in leukemic stem and progenitor cells in pediatric AML. Genome Biol. 24 (1), 199. 10.1186/s13059-023-03031-7 37653425 PMC10472599

[B151] Zhang Y. Park M. Ghoda L. Y. Zhao D. Valerio M. Nafie E. (2024). IL1RAP-specific T cell engager depletes acute myeloid leukemia stem cells. J. Hematol. Oncol. 17 (1), 67. 10.1186/s13045-024-01586-x 39143574 PMC11325815

[B152] Zhang Z. Wilhelm M. Sieber I. Döhner H. Feuring M. (2025). Development of a 29‐Color single‐tube full spectrum flow cytometry assay for the detection of measurable residual disease and leukemic stem cells in acute myeloid leukemia. Cytom. Part A 107, 597–613. 10.1002/cyto.a.24958 40937645

[B153] Zhou J. Wu H. Li B. Lv L. Zhu S. Liang A. (2025). CD69 predicts prognosis through immune cell infiltration and decitabine treatment response in acute myeloid leukemia. Transl. Cancer Res. 14 (2), 865–880. 10.21037/tcr-24-1550 40104698 PMC11912086

